# A88 PATIENT-CENTRIC VALIDATION OF A PROVINCE-WIDE COLONOSCOPY REFERRAL SHEET DETERMINING WAIT-TIME PROCEDURAL ALLOCATION

**DOI:** 10.1093/jcag/gwae059.088

**Published:** 2025-02-10

**Authors:** A Barkun, S Aprikian, C Hansen-Barkun, D Kim, G Milky, J Beauchesne-Blanchet, M Martel, C Menard, D von Renteln

**Affiliations:** McGill University, Montreal, QC, Canada; McGill University, Montreal, QC, Canada; Research Institute of the McGill University Health Centre, Montreal, QC, Canada; McGill University, Montreal, QC, Canada; Research Institute of the McGill University Health Centre, Montreal, QC, Canada; Research Institute of the McGill University Health Centre, Montreal, QC, Canada; Research Institute of the McGill University Health Centre, Montreal, QC, Canada; Universite de Sherbrooke, Sherbrooke, QC, Canada; Universite de Montreal, Montreal, QC, Canada

## Abstract

**Background:**

The widespread use of a standardized, validated province-wide colonoscopy referral form (PCRF), regrouping mutually exclusive indications into suggested priority wait time categories has allowed for a more comprehensive, patient-centric assessment of routine colonoscopy practice

**Aims:**

To better characterize colonoscopic finding yields based on specific clinical indications (PCRF) determined by patient symptoms’ questionnaire-generated indications rather than referring physician-based indications. We also attempt further validation of the PCRF.

**Methods:**

Prospective cohort of consecutive adult patients from two hospitals. Information collected from PCRF (therefore referring physician assessment) but also additional symptoms from a patient questionnaire. The primary outcome was the colonoscopy findings. Descriptive and inferential statistics and multivariable regression analyze predictive modeling of different indications and symptoms.

**Results:**

Overall, 5979 patients (mean age 59.2±14.2years, 49.8% female, mean BMI 26.7±5.2) were included from June 2022 to February 2024. Duration between colonoscopy referral and colonoscopy (days) was 266.7 ± 405.9 (median=138 days). Excluding diverticulosis and non-bleeding hemorrhoids, 41.3% of patients had clinically significant lesions and 0.9% adenocarcinomas. The main indication according to patients was surveillance of polyps (IN13 - 35.7%), surveillance for significant family history (IN21 - 21.2%), IBD surveillance (IN15 - 11.7%). Based on additional questions of the patient questionnaire that did not correspond to any indication on the PCRF, 70.0% had no additional symptoms while 13.3% experienced abdominal pains, and 11.1% cramping, with 4.8% reporting weight loss. Between-group comparisons for adenocarcinoma and clinically significant lesions are described in figure 1. Models with the best fit for predicting adenocarcinoma were associated with the combinations of age>40, and presence of IN2, IN5, IN6 and IN17 with OR=7.74 (4.45; 13.46) (or OR=6.46 (2.78; 15.14) for age>60). Clinically significant lesions were associated with the combinations of age>60, IN5, IN17 and IN13 with OR=2.05 (1.83; 2.90).

**Conclusions:**

This exercise has allowed further validation of recently adopted provincial changes in PCRF priorities attributed to some indications. Addition of symptoms not captured in PCRF indications did not improve prediction using existing indications in multivariable modelling. The use of multiple PCRF indication in allocating waiting priorities rather than choosing the sole perceived most urgent one (which is how the PCRF is used) now requires prospective validation.

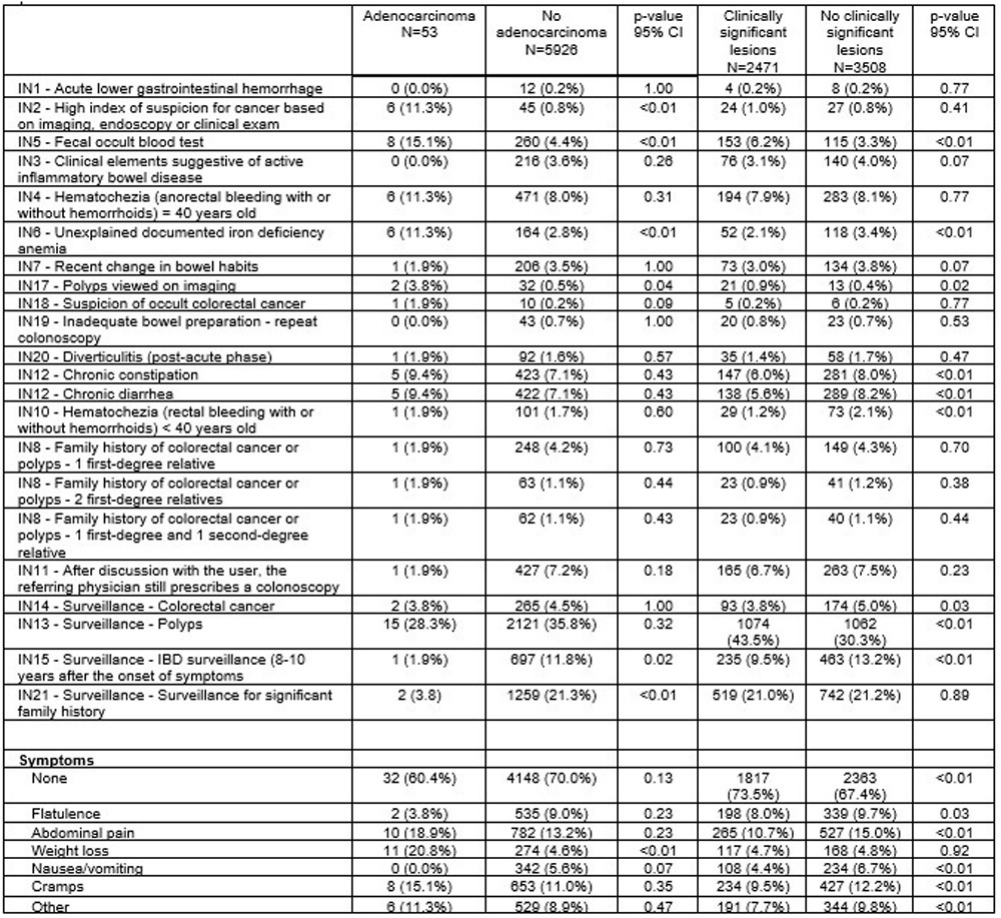

**Funding Agencies:**

CPAC and MSSS

